# Editorial: Foodborne Enterobacteriaceae of Animal Origin

**DOI:** 10.3389/fcimb.2021.772359

**Published:** 2021-09-28

**Authors:** Fangkun Wang, Wanjiang Zhang, Dongyan Niu

**Affiliations:** ^1^ Shandong Provincial Key Laboratory of Animal Biotechnology and Disease Control and Prevention, College of Veterinary Medicine, Shandong Agricultural University, Tai’an, China; ^2^ State Key Laboratory of Veterinary Biotechnology, Harbin Veterinary Research Institute, Chinese Academy of Agricultural Sciences, Harbin, China; ^3^ Ecosystem and Public Health, Faculty of Veterinary Medicine, University of Calgary, Calgary, AB, Canada

**Keywords:** foodborne Enterobacter bacteria, animal origin, epidemic characteristics, drug resistance, pathogenic mechanism, control measure

## Introduction

The main pathogens responsible for livestock and poultry products causing foodborne epidemics in people include *Salmonella*, *Campylobacter jejuni*, *Escherichia coli*, *Shigella*, *Proteus*, etc. These Enterobacteriaceae are important zoonotic foodborne bacteria capable of endangering human health. A high ongoing prevalence and occasional outbreaks of these bacteria cause global morbidity and mortality, leading to huge economic losses. The first objective of this Research Topic is to estimate the prevalence of resistance to common antimicrobial agents amongst Enterobacteriaceae isolated from livestock and poultry. The second objective is to advance food safety from farm to fork by understanding human health risks potentially posed by foodborne pathogens of animal origin, as well as to study pathogenic mechanisms and develop innovative strategies to minimize food safety risk. This topic contains 11 research and 2 review articles, covering a variety of topics, including epidemic characteristics of drug resistance, pathogenic mechanisms, and novel control measures.

## Epidemic Characteristics of Drug Resistance of Foodborne Enterobacteriaceae

The global increase in antibiotic-resistant bacteria is a worldwide concern for human and animal health. In this topic, five research papers report epidemic characteristics of drug resistance in foodborne Enterobacteriaceae. Song et al. and Xu et al. investigated epidemiological characteristics of salmonellosis in breeder chickens in 10 Chinese provinces; among the *Salmonella* prevalent on poultry breeder farms in various provinces, *S. pullorum* and *S. enteritidis* continue to be major etiological agents. Most *Salmonella* strains isolated were multidrug-resistant (MDR), presenting a serious problem for animal and human. Therefore, it is necessary to monitor, control, and rationalize antimicrobial use on chicken farms to limit resistance against antimicrobial agents. These findings provide current information regarding the prevalence of antibiotic-resistant *Salmonella* on breeding poultry farms and inform the long-term goal of eradicating salmonellosis in China. Cui et al. focused on epidemiological characteristics of *Salmonella* in chicken flocks fed antibiotic-free diets. Herein, they investigated and compared *Salmonella* infections in three Chinese native breeders fed antibiotic-free diets and one conventional breeder, the Bairi chicken, in 2019. Antibiotic resistance rates and MDR rates in three chicken breeds fed antibiotic-free diets were significantly lower than that from a conventional Bairi chicken farm. In the article by Zhao et al., characteristics of Salmonella infections in the context of chicken mortality at hatching in Shandong were explored. *Salmonella* isolates with four sequence types (STs) were recovered from 6.7% of these embryos, with ST26 being the most prevalent; they inferred that *Salmonella* infections may be an important cause of chicken embryo mortality. In the article by Su et al., antibiotic resistance, molecular profiles, and intrinsic relationships of Shiga toxin-producing *E. coli* (STEC) O157:H7 isolates from cattle farms and abattoirs in Xinjiang were investigated. The *bla_CTX−M_* gene encoding extended spectrum β-lactamases (ESBL) was first detected in STEC O157:H7 from cattle in Xinjiang. Furthermore, this gene was transferable under experimental conditions, highlighting potential dissemination of β-lactam resistance from cattle.

## Pathogenic Mechanisms of Foodborne Enterobacteriaceae

In this topic, two research papers and one review focused on STEC. Shiga toxin (Stx) is the main virulence factor of STEC, with Stx2 being most frequently linked to severe illness. Ruminants, especially cattle, are the main reservoir of STEC. Pan et al. identified the correlation between *stx2* expression and expression of bovine host immune genes, i.e., *stx2* expression may be associated with expression of genes involved in B-cell proliferation and lymphotoxin beta production. Furthermore, they used artificial intelligence-based approaches to identify potential gene markers for *stx2* expression in cattle, shedding light on utility of host immune genes for predicting STEC colonization. In the article by Wang et al., microRNAomes of cattle intestinal tissues revealed possible miRNA-regulated mechanisms involved in *E. coli* O157 fecal shedding. The identified miRNAs and their functions increase understanding of molecular mechanisms regulating colonization of this foodborne pathogen *in-vivo*, which is vital to reduce cattle that are STEC O157 supershedders. Hwang et al. also provided a comprehensive review on STEC infection strategies, including mode of transmission of STEC and effects of Stx in humans and animals, STEC attachment and pathogenicity in the intestinal environment, and correlations between antimicrobial resistance (AR) and increased toxin gene expression in STEC.

Two papers focused on drug resistance mechanisms of foodborne Enterobacteriaceae. In the article by Luo et al., common ESBL encoding genes *bla_NDM_* were profiled from chromosomal mobile genetic elements (MGE) of *Providencia rettgeri*, *P. mirabilis*, and *Klebsiella pneumoniae.* This study dealt with an extensive sequence comparison of 12 chromosomal genetic elements, including known integrative and conjugative elements (ICE), integrative and mobilizable elements (IME), *bla_NDM_*-carrying transposons and newly sequenced *bla_NDM_*-carrying ones. They determined that these 12 genetic elements encoded >51 resistance genes involved in resistance to 18 categories of antibiotics and heavy metals. In addition, 8 novel *bla_NDM_*-carrying MGE were identified. This study provided deeper genetic insights into chromosomal integration of *bla_NDM_*-carrying genetic elements in Enterobacteriaceae. Tran et al. identified MDR *E. coli* and *Salmonella* from broiler chicken production in Alberta, Canada, providing insights into potential horizontal gene transfer (HGT) events of AR genes between *E. coli* and *Salmonella* in broiler chickens. Moreover, certain MDR *E. coli* and *Salmonella* exhibited ESBL phenotypes encoded by transferable plasmids, suggesting HGT-associated MDR dissemination may readily occur in broiler chicken production.

Enteropathogenic *E. coli* (EPEC) continue to be the leading bacterial cause of infant diarrhea worldwide. Ledwaba et al. optimized a murine infection model that uses depletion of intestinal microbiota by antibiotics in weaned mice, enabling enhanced bacteria colonization; this will facilitate exploration of mechanisms involved in EPEC pathogenesis and development of vaccines or interventions.

The Mycotoxin Deoxynivalenol (DON) is a major health concern in poultry production, as it targets epithelial cells of the gastrointestinal tract and contributes to a loss of epithelial barrier function. Here, Ruhnau et al. reported that DON promoted *C. jejuni* multiplication in the intestine of broiler chickens, with consequences for bacterial translocation and gut integrity. Co-exposure of broilers to DON and *C. jejuni* can potentiate gut permeability, promoting *Campylobacter* colonization and its translocation across the intestinal epithelium.

## Novel Control Measures for Foodborne Enterobacteriaceae Bacterial Diseases

To reduce both prophylactic and sub-therapeutic use of antibiotics, many studies have focused on alternatives with similar antibacterial and animal growth-promoting effects without depressing beneficial microbiota and minimizing development of antimicrobial resistance in animals. These alternatives include antimicrobial peptides (AMPs), probiotics, phytogenic compounds, etc. Protective effects of natural AMPs in food animals (i.e., swine, cattle and poultry), factors limiting the efficacy of these AMPs, and mitigating strategies were comprehensively reviewed by Fasina et al. It was concluded that AMP may partially replace conventional antibiotics in food animal production, thereby improving quality and microbiological safety of animal meat and egg products for human consumption.

## Conclusions

The articles in this Research Topic enriched knowledge on epidemic characteristics of drug resistance and their genetic determinants, pathogenic mechanisms, and novel control measures of foodborne Enterobacteriaceae. This group contains a wide range of Gram-negative bacteria that commonly originate from food animals and are capable of entering the food supply chain, causing food poisoning and even life-threating illness. The complexity and degree of resistance possessed by these foodborne Enterobacteriaceae to clinically important antibiotics is vast and emerging. Therefore, there is an urgent need to address the AMR crisis from food animal origin by employing a “One Health” concept. There is a need for a broad range of research, including: monitoring management practices that promote AMR transfer at the animal, environment and human interface; understanding molecular mechanisms underlying persistence and transmission of foodborne pathogens in the animal food supply chain using multi-omics approaches; and mechanistic insights into their pathogenicity and AMR characteristics. As a final comment, the editors of this topic expresses their deep appreciation to all authors and reviewers for their great contributions to the collection.

## Author Contributions

FW, WZ, and DN edited the Research Topic of Foodborne Enterobacteriaceae of Animal Origin, wrote the manuscript, and approved it for publication. All authors contributed to the article and approved the submitted version.

## Conflict of Interest

The authors declare that the research was conducted in the absence of any commercial or financial relationships that could be construed as a potential conflict of interest.

## Publisher’s Note

All claims expressed in this article are solely those of the authors and do not necessarily represent those of their affiliated organizations, or those of the publisher, the editors and the reviewers. Any product that may be evaluated in this article, or claim that may be made by its manufacturer, is not guaranteed or endorsed by the publisher.

